# Genetic architecture of cowpea domestication: QTL mapping and comparison shed new light on the dual domestication events

**DOI:** 10.1093/g3journal/jkaf248

**Published:** 2025-10-17

**Authors:** Demba Dramé, Amy Bodian, Joel Romaric Nguepjop, Hodo-Abalo Tossim, Diarietou Sambakhe, Maguette Seye, Yvette Rachelle Djiboune, Romiel Badji, Jean Francois Rami, Diaga Diouf, Daniel Fonceka

**Affiliations:** Centre d’Etude Régional pour l’Amélioration de l’Adaptation à la Sécheresse (CERAAS)/Institut Sénégalais de Recherches Agricoles (ISRA), BP3320 Thiès, Senegal; Laboratoire Campus de Biotechnologies Végétales, Département de Biologie Végétale, Faculté des Sciences et Techniques, Université Cheikh Anta Diop (UCAD), Dakar-Fann, Dakar 10700, Senegal; Centre d’Etude Régional pour l’Amélioration de l’Adaptation à la Sécheresse (CERAAS)/Institut Sénégalais de Recherches Agricoles (ISRA), BP3320 Thiès, Senegal; Centre d’Etude Régional pour l’Amélioration de l’Adaptation à la Sécheresse (CERAAS)/Institut Sénégalais de Recherches Agricoles (ISRA), BP3320 Thiès, Senegal; Centre de Coopération Internationale en Recherche Agronomique Pour le Développement (Cirad), UMR AGAP, Montpellier 34398, France; CIRAD, INRAE, AGAP, University Montpellier, Institut Agro, Montpellier, France; Centre d’Etude Régional pour l’Amélioration de l’Adaptation à la Sécheresse (CERAAS)/Institut Sénégalais de Recherches Agricoles (ISRA), BP3320 Thiès, Senegal; Centre d’Etude Régional pour l’Amélioration de l’Adaptation à la Sécheresse (CERAAS)/Institut Sénégalais de Recherches Agricoles (ISRA), BP3320 Thiès, Senegal; Centre d’Etude Régional pour l’Amélioration de l’Adaptation à la Sécheresse (CERAAS)/Institut Sénégalais de Recherches Agricoles (ISRA), BP3320 Thiès, Senegal; Centre d’Etude Régional pour l’Amélioration de l’Adaptation à la Sécheresse (CERAAS)/Institut Sénégalais de Recherches Agricoles (ISRA), BP3320 Thiès, Senegal; Centre de Recherches Agronomiques (CNRA) de Bambey, Institut Sénégalais de Recherches Agricoles (ISRA), BP53 Bambey, Senegal; Centre de Coopération Internationale en Recherche Agronomique Pour le Développement (Cirad), UMR AGAP, Montpellier 34398, France; CIRAD, INRAE, AGAP, University Montpellier, Institut Agro, Montpellier, France; Laboratoire Campus de Biotechnologies Végétales, Département de Biologie Végétale, Faculté des Sciences et Techniques, Université Cheikh Anta Diop (UCAD), Dakar-Fann, Dakar 10700, Senegal; Centre d’Etude Régional pour l’Amélioration de l’Adaptation à la Sécheresse (CERAAS)/Institut Sénégalais de Recherches Agricoles (ISRA), BP3320 Thiès, Senegal; Centre de Coopération Internationale en Recherche Agronomique Pour le Développement (Cirad), UMR AGAP, Montpellier 34398, France; CIRAD, INRAE, AGAP, University Montpellier, Institut Agro, Montpellier, France

**Keywords:** cowpea (*Vigna unguiculata*), domestication-related traits (DRT), dual domestication, SNP, quantitative trait loci

## Abstract

Understanding the genetic basis of domestication-related traits (DRTs) is crucial for crop improvement. In this study, we developed an interspecific backcross population by crossing the elite cowpea variety Sam with a wild accession of *Vigna unguiculata* var. *spontanea* from Senegal. Using a mid-density single nucleotide polymorphism panel, we constructed a high-quality genetic linkage map consisting of 1,046 polymorphic markers spanning 1,131.6 cM across 11 chromosomes and used it as a framework for dissecting the genetic architecture of key DRTs. Over 2 consecutive years, we identified 65 quantitative trait loci (QTLs) associated with 17 key domestication traits, with 73.8% of these QTLs consistently detected across both years. Notably, we observed a significant clustering of domestication-related QTLs within 4 major genomic regions on chromosomes Vu01, Vu03, Vu08, and Vu09, particularly for organ size and phenological traits. The co-location of QTLs for traits such as pod shattering, growth habit, and flowering time suggests pleiotropy or potential co-selection of linked genes during domestication. Furthermore, our findings support the hypothesis of 2 independent domestication events in cowpea, as evidenced by similarities as well as differences in QTL regions between our study and previous reports. We hypothesized that common as well as different loci may have been selected during the 2 independent domestication events of cowpea, paralleling the dual domestication in common beans. While wild cowpea species contributed limited major-effect QTLs for yield-related traits, they remain an essential reservoir of genetic diversity, particularly for pest and disease resistance. These insights enhance our understanding of cowpea domestication and offer valuable genetic resources for breeding programs.

## Introduction

Cowpea [*Vigna unguiculata* (L.) Walpers] is a diploid species of the Fabaceae family, with a chromosome count of 2n = 2x = 22 (Pasquet 1999; Liang et al. 2024), and an estimated nuclear genome size of 640.6 Mbp (Lonardi et al. 2019). Cowpeas, which originated in Africa, have become an essential crop cultivated across the globe (Herniter et al. 2020; Liang et al. 2024). As a crucial source of protein in sub-Saharan Africa, cowpeas significantly contribute to food security (Herniter et al. 2020) and bolster nutritional stability in developing countries (Wu et al. 2024). This legume not only provides nutritious seeds for human consumption and valuable fodder for livestock but also enhances soil fertility through its natural ability to fix nitrogen by establishing symbiotic association with *Bradyrhizobium* (Wortman and Dawson 2015; Mulugeta et al. 2016; Samireddypalle et al. 2017; Boukar et al. 2020; Herniter et al. 2020; Liang et al. 2024; Ongom et al. 2024). Compared to other commonly cultivated legume species, cowpea requires less water, is heat tolerant, and achieves appreciable yields under drought prone environment (Herniter et al. 2020).

*V. unguiculata* has morphologically and genetically diverse gene pool, consisting of several wild taxa and cultivated varieties (Ba et al. 2004). Recent taxonomic revisions have classified cowpea into 10 perennial subspecies and 1 annual subspecies, subsp. *unguiculata,* which included the cultivated form (*V. unguiculata* var. *unguiculata*) and its wild progenitor (*V. unguiculata* var. *spontanea*) (Pasquet 1997; Coulibaly et al. 2002; Maxted et al. 2004). The cultivated subspecies *unguiculata* var. *unguiculata* has been divided into 5 cultigroups: *Unguiculata*, *Biflora*, *Melanophthalmus*, *Sesquipedalis*, and *Textilis* (Pasquet 1998). While the cultivated cowpeas are all annual, their wild relatives include both perennial and annual forms (Pasquet et al. 1997).

Both West Africa and East Africa have been proposed as centers of domestication for cowpea (Coulibaly et al. 2002; Ba et al. 2004; Huynh et al. 2013; Xiong et al. 2016). Remarkably, the cultivated varieties of cowpea in these areas show a close genetic relationship to their native wild relatives, underscoring the importance of these regions in the history and development of this crop. However, the question of whether cowpea underwent a single or 2 domestication events is still controversial. Cowpea domestication resulted in significant changes from its wild ancestors, including resistance to pod shattering, increased in seed, pod and leaf sizes, loss of perennial growth, and alteration in flower color and scent (Lo et al. 2020). The suite of morphological and physiological changes that have arisen through plant evolution under human selection is collectively referred to as the “domestication syndrome” (Harlan 1992; Lo et al. 2018).

The development of genomic resources made it possible to understand the genetic control of domestication-related traits (DRTs) in crops. For instance, quantitative trait loci (QTLs) and genes responsible for DRTs have been identified in maize (Lauter and Doebley 2002), pearl millet (Poncet et al. 2002), wheat (Peng et al. 2003; Ross-Ibarra 2005), and zombi pea (Amkul et al. 2020), among others. In legume species, the genetic control of DRTs is primarily mono or oligogenic. Shattering resistance is governed by 1 major gene in soybean (Funatsuki et al. 2008), pea (Weeden 2007), and bean (Parker et al. 2020), while it is controlled by 2 recessive genes (tardus and lentus) in lupine (Gladstones 1967). Boersma et al. (2009) subsequently mapped and developed molecular markers specifically linked to the tardus gene. Similar patterns are observed for seed dormancy, growth habit and flowering time (reviewed by Ambika et al. 2022). The genetic basis of DRTs has been studied in cowpea, specifically in 2 subspecies: *V. unguiculata* ssp. *sesquipedalis* (yardlong bean, also known as vegetable cowpea) and *V. unguiculata* ssp. *unguiculata* (grain-type cowpea). In yardlong bean, Xu et al. (2011) reported that flower and seed coat colors exhibited monogenic inheritance, with the genes controlling these 2 traits located closely together, in a mapping population derived from a cross between a landrace and a cultivated variety from China. Kongjaimun et al. (2012) conducted a study using a cross between a yardlong bean variety from Sri Lanka and TVnu-457, a wild cowpea (*V. unguiculata* ssp. *unguiculata* var. *spontanea*) from Mali. They mapped 153 QTLs—ranging from 1 to 11 QTLs per trait across 24 DRTs—among which 32 QTLs were considered major, explaining more than 10% of the phenotypic variance.

In contrast, the mapping of QTLs for DRTs in grain-type cowpea using biparental mapping populations has been somewhat limited. Ubi et al. (2000) used Random Amplified Polymorphic DNA (RAPD) markers and a recombinant inbred line (RIL) population derived from the cross between IT84S-2246-4, an improved line, and TVNu110-3A (*V. unguiculata* ssp. *dekindtiana* var. *pubescence*), a wild relative sourced at the International Institute of Tropical Agriculture (IITA), to identify 88 QTLs for 12 DRTs. Andargie et al. (2014) identified 10 QTLs associated with seed weight and pod fiber layer thickness using a RIL population derived from a cross between 524B, a cultivated variety from California, and a unique wild perennial cowpea (*V. unguiculata* subsp*. unguiculata* var*. spontanea*) from coastal Kenya. In a detailed study, Lo et al. (2018) identified 16 QTLs for 9 DRTs using an RIL population developed from a cross between IT99K-573-1-1 (also known as Sampea14), an early-maturing cowpea variety from Nigeria, and TVNu-1158, a wild perennial cowpea collected in the Republic of Congo. This study also proposed candidate genes associated with key traits such as pod-shattering resistance, flowering time, seed size, peduncle length, and flower color. A comparison of QTLs between the 2 studies revealed that none of the loci associated with 3 critical traits—pod shattering, seed size, and flower color—overlapped with those reported by Andargie et al. (2014). Lo et al. (2018) concluded that their identified QTLs were novel. However, the role of germplasm origin in shaping these findings has not been discussed. The discrepancy in QTL regions between the 2 studies may stem from the different geographical origins of the parental lines used. This suggests that distinct loci may have been selected during independent domestication events of cowpea. These findings also raise the question of the extent to which domestication QTLs and genes coincide or diverge in interspecific populations that include parental lines from different geographical origins.

In this study, we established a backcross population by crossing an elite cowpea cultivar with a wild accession, both sourced from Senegal. Our primary objective was to delve into the genetic foundations of cowpea domestication. We identified several key QTLs linked to this process and conducted a comparative analysis of these regions with findings from other research. Our results offer significant insights into the genetic mechanisms underlying domestication in cowpeas.

## Materials and methods

### Plant materials and population development

**Fig. 1.**
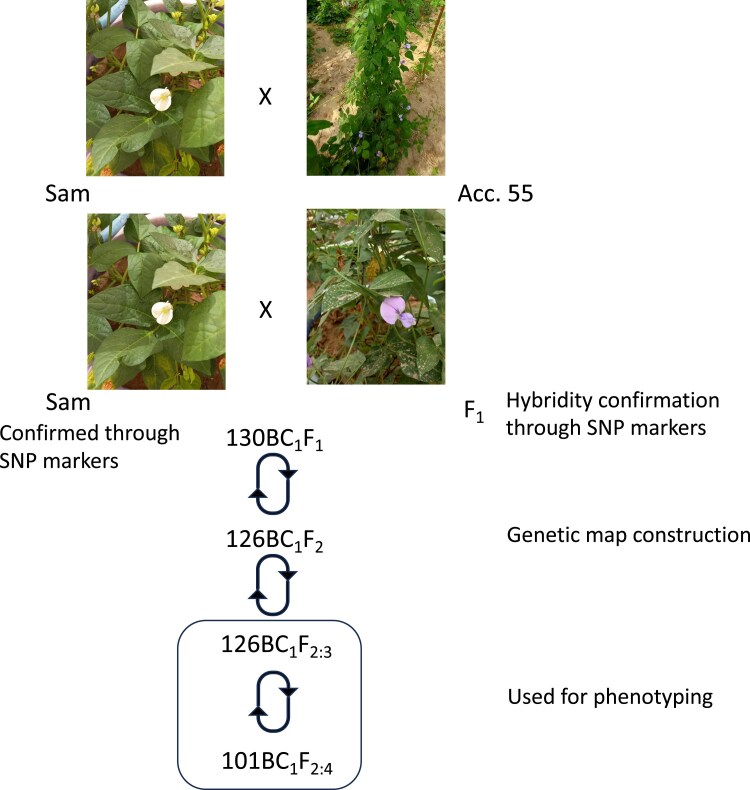
Breeding scheme used for the development of the interspecific QTL mapping population. The cultivated parent Sam was crossed with the wild accession Acc. 55 to produce F_1_ hybrids, which were backcrossed to Sam to generate BC_1_F_1_ progeny. F_1_ hybrids and BC_1_F_1_ plants were confirmed using SNP markers. The BC_1_F_2_ plants were used to construct the genetic map. BC_1_F_2:3_ and BC_1_F_2:4_ generations were subsequently developed and used for phenotypic evaluation.

A population of 126 BC_1_F_2_ was produced from a cross between the improved cultivated cowpea variety Sam (*V. unguiculata* var. *unguiculata*), and a wild species, *V. unguiculata* var. *spontanea*. The cultivated variety used as female parent is a pure line with erect growth habit, white flowers and nonshattering pods, registered in Senegal (Cissé 2015). The wild cowpea accession (acc. 55) used as male parent was collected in Senegal, Fatick region, Mbouloum village (latitude 13°89.78 and longitude 16°35.86) (Sarr et al. 2021). It has a climbing growth habit, violet flowers and pods shattering characteristics (Dramé et al. 2023). The BC_1_F_1_ generation was developed, by backcrossing a single F_1_ hybrid plant to the cultivated cowpea recurrent Sam parent. BC1F1 plants were checked for true hybridity using single nucleotide polymorphism (SNP) markers, and 126 BC_1_F_1_ were selected. The 126 BC_1_F_1_ were grown in plastic pots, and allowed to self-pollinate to produce the BC_1_F_2_ generation (Fig. 1). Each BC_1_F_2_ plant was derived from the selfing of a single BC1F1 seed. Therefore, 126 BC_1_F_2_ were used to produce 126 BC_1_F_2:3_ and 101 BC_1_F_2:4_ families that were used for field evaluation. All stages of the population development were performed under shadehouse conditions at the Centre d’Etude Régional pour l’Amelioration de l’Adaptation à la Sécheresse (CERAAS), Thiès, Senegal between 2021 and 2022.

### Phenotyping

#### Field preparation

The experiments were conducted during 2 consecutive rainy seasons (2023 and 2024) at the Centre National de Recherche Agronomique (CNRA) in Bambey (14.42° N and 16.28W°), Senegal. In this research station, the soil is ferruginous, with 90% sand content, and low clay content (<10%). Forty-five days before sowing, the field was plowed to eliminate weeds. One hundred and fifty kg/ha of organic fertilizer and 1 t/ha of mineral fertilizer N-P-K (6-20-10) were added 4 wk and 3 d before sowing, respectively. The field was kept manually weed-free before sowing and throughout the experiment.

#### Experimental design

Alpha-lattice designs were employed for the field trials. In 2023, 126 BC_1_F_2:3_ families and the cultivated parent (which was repeated twice) were tested across 3 replications. Each replication consisted of 16 blocks, with each block containing 8 families. In 2024, 101 BC_1_F_2:4_ families along with the cultivated parent (repeated 3 times) were evaluated in 3 replications. Each replication included 13 blocks, with each block containing also 8 families. In each experiment, the plants were arranged in rows, with 10 pockets per row. The spacing was 70 cm between plants and 75 cm between rows. 3 seeds were sown in each hole, and 2 wk after sowing, the plants were thinned to 2 per pocket. Consequently, in a given replication, each family was represented by a single row of 20 plants.

#### Trait evaluation

A total of 17 DRTs were evaluated in the trials conducted in 2023 and 2024. The variables are listed in Table 1 in 3 categories: morphology, phenology, and yield components.

**Table 1.** List of measured traits and associated methods and scales.

Category	Trait name	Code	Method	Measurement stage	Scale
Morphology	Flower color	Flc	Visual scoring	Flowering	0 = white, 1 = intermediate, and 3 = violet
	Growth Habit	Gh	Visual scoring	Maturity	0 = erect, 1 = intermediate, and 2 = climbing
	Pod shattering	Pdsh	Visual scoring	Maturity	0 = resistant to shattering and 1 = nonresistant to shattering
	Seed coat color	Scc	Visual scoring	After harvest	0 = non-White, 1 = White
	Terminal leaf length	Tlfl	Measured using a measuring tape on 10 plants	6 wk after sowing	cm
	Terminal leaf width	Tlfw	Measured using a measuring tape on 10 plants	6 wk after sowing	cm
	Main stem length	MSL	Measured using a ruler on 10 plants	Maturity	cm
Phenology	Time to first flower	DFw	Recorded days from sowing until the appearance of the first flower on a plot	NA	Number of days
	Time to 50% flowering	T50fw	Recorded days from sowing to 50% of the plants flowered	NA	Number of days
	Time to first mature pod	DRp	Recorded days from sowing until the appearance of first the pod	NA	Number of days
	Time to 95% pod maturity	T95Rp	Recorded days from sowing to 95% of mature pods in a plot	NA	Number of days
Yield components	Pod length	Pdl	Measured using a measuring tape on 30 pods	After harvest	mm
	Pod width	Pdw	Measured using a measuring tape on 30 pods	After harvest	mm
	Seed length	Sdl	Measured using a measuring tape on 30 seeds	After harvest	mm
	Seed width	Sdw	Measured using a measuring tape on 30 seeds	After harvest	mm
	Dry haulm weight	Hw	Weighed with precision scale	After harvest	g
	100-seed weight	HSdwg	Weighed with precision scale	After harvest	g

#### Data analysis

For each quantitative trait, the mean, standard error and range were calculated, and phenotype frequency distributions were examined in the BC_1_F_2:3_ and BC_1_F_2:4_ populations and the cultivated parent using R Software version 4.1.2 (R Core Team 2014). An analysis of variance (ANOVA) was performed with the aov function for 2023 and 2024 data to estimate genotype, replication, and block effects using the model:


$$Y_{\mathrm{ijk}}=\mu +G_{i}+R_{j}+B_{\mathrm{jk}}+\varepsilon_{\mathrm{ijk}}$$

where Y_ijk_ is the observed trait value, μ is the intercept, G_i_ is the genotype effect, R_j_ is the replication effect, B_jk_ is the block within replication effect, and $\varepsilon_{\mathrm{ijk}}$ is the residual error. Normality of the residuals was assessed using the Shapiro–Wilk test. Least square means (LSMEANS) were extracted using the emmeans package (Lenth 2025) and used for QTL analyses. Trait phenotypic correlation was computed using the corrplot package (Wei and Simko 2024). All statistical analyses and graph construction were conducted in R Software.

Estimates of broad-sense heritability were calculated as follows:


$$h^{2}=\frac{\sigma_{G}^{2}}{{(\sigma }_{G}^{2}+\sigma_{E}^{2})}\;\mathrm{with}\;\sigma_{G}^{2}=\frac{\left(MS_{G}-MS_{E}\right)}{r}\;\mathrm{and}\;\sigma_{E}^{2}=MS_{E}$$

where $\sigma_{G}^{2}$ is the genotypic variance, $\sigma_{E}^{2}$ the residual variation, MS_G_ and MS_E_ the genetic and residual mean squares, and r the number of replications.

### Genetic map construction

Young leaves of the 126 BC1F2 individuals were sampled and sent to Intertek for genotyping using the mid-density SNP panel developed by Ongom et al. (2024). The genotyping data was curated by removing monomorphic SNPs and those with more than 20% missing data. Polymorphic SNPs between the cultivated and wild parents were selected to build the linkage groups using the following parameters: minimum Logarithm of the Odds (LOD) of 4 and maximum recombination fraction of 0.3. Kosambi's mapping function as described by Kosambi (1943) was employed to convert the recombination fractions into genetic map distances measured in centiMorgans (cM). The linkage Groups were named and oriented from Vu01 to Vu11 as indicated in Ongom et al. (2024).

### QTLs analysis

The R/qtl package (Broman et al. 2003) was used for the QTL identification. Genotype probabilities were first estimated at each 1 cM interval using the “calc.genoprob” function.

Multiple Interval Mapping (MIM) using the multiple QTL model selection approach described by Broman and Speed (2002), was used to detect QTLs. The *stepwiseqtl* function was used for forward/backward model selection using Haley–Knott regression, considering only additive QTL models, and with a maximum number of 8 QTLs. The main effect LOD penalty was computed for each trait from 500 permutations of a 2 QTLs genome scan using the *scantwo* function with a significance level of 0.05. A simple interval mapping (SIM) scan was also performed using the *scanone* function. Reported QTLs were those that were detected using MIM, SIM or both methods. For the Gh and Scc traits that showed binary distributions, the binary model proposed by (Xu and Atchley 1996) was used in replacement of the usual normal model.

The LOD value and percentage of phenotypic variance for each QTL were estimated from a drop-one analysis comparing the full model to each submodel with 1 QTL dropped. The additive effect of each QTL was estimated as half the difference between the homozygous classes of wild and cultivated parents. A positive additive effect thus corresponds to a QTL for which the wild parent brings a positive effect. Confidence intervals of all QTLs were determined using the approximate Bayes credible interval method implemented in the R/qtl package (Broman et al. 2003; Broman and Sen 2009)

The graphical representation of the QTLs was obtained using Spidermap software (Rami, unpublished).

### QTL comparison

QTL comparison was conducted using data from Lo et al. (2018) and Andargie et al. (2014). For the comparison with Lo et al. (2018) , we searched for the SNP peak physical positions at each QTL, referring to the 51,128 Cowpea iSelect Consortium Array (Muñoz-Amatriaín et al. 2017). Additionally, we performed BLAST searches of the marker sequences (forward and reverse sequences) from the simple sequence repeat (SSR) peaks at each QTL reported in Andargie et al. (2014) against the reference genome sequence available at www.phytozome.net (Goodstein et al. 2012). These searches were carried out using BLAST version 2.16, which can be accessed at https://sequenceserver.legumeinfo.org/ (Berendzen et al. 2021).

## Results

### Trait variability and heritability

The phenotypic values were normally distributed both for trials carried out in 2023 and 2024 (Supplementary Fig. 1). The recurrent parent and population mean, standard error, and range as well as the broad-sense heritabilities (h^2^) of each trait are shown in Table 2.

**Table 2.** Summary statistics and heritability analysis in 2023 and 2024.

Code	2023					2024				
	Sam	Mean	SE	Range	h^2^	Sam	Mean	SE	Min to Max	h^2^
Tlfl	10	10.87	0.4	7.44 to 14.61	0.66	10.7	11.61	0.41	8.83 to 15.3	0.6
Tlfw	4.85	5.21	0.29	2.83 to 8.01	0.75	5.52	5.22	0.39	2.56 to 8.38	0.52
MSL	81.5	84.82	17.21	15.25 to 239.80	0.38	106.2	117.88	16.02	17.33 to 253.2	0.71
DFw	38	40.91	0.98	31 to 88	0.98	38	38.3	1.46	31 to 88	0.9
T50fw	40.33	42.35	0.79	35.00 to 95.00	0.89	41	42.9	1.59	35 to 93	0.89
DRp	53.3	54.48	0.82	48 to 109	0.96	53.67	52.86	1.17	46 to 105	0.89
T95Rp	60	62.23	1.14	55.00 to 117.00	0.89	60	62.27	1.3	55 to 115	0.93
Pdl	171.28	134.4	0.54	75.67 to 192.00	0.75	188	144.1	0.76	91 to 191	0.55
Pdw	10.49	8.43	0.29	5.67 to 10	0.58	9	8.15	0.03	5.77 to 10	0.62
Sdl	9.65	8.04	0.22	5.23 to 10.41	0.86	9.38	8.13	0.27	5.69 to 10.84	0.74
Sdw	6.41	5.33	0.13	3.63 to 6.55	0.73	6.46	5.42	0.13	4.39 to 6.6	0.65
Hw	16.12	25.38	5.03	5.25 to 58.71	0.3	75	97.91	19.24	20 to 373	0.56
HSdwg	24.92	15.26	0.93	7.46 to 27.27	0.69	21.57	14.58	1.1	7.87 to 29.80	0.6

SE, standard error of mean; h^2^, broad-sense heritability.

**Fig. 2.**
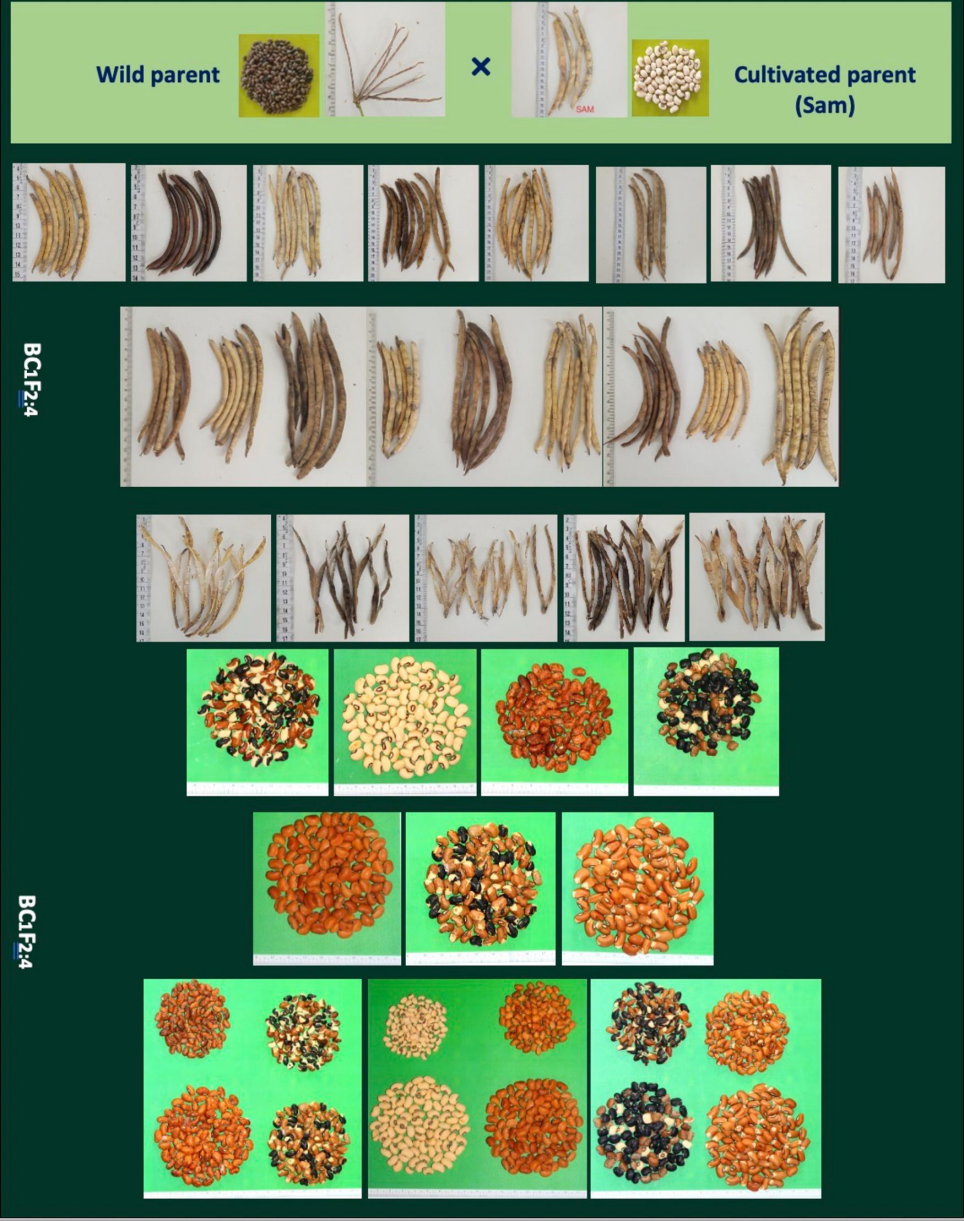
Phenotypic variation observed among BC_1_F_2:4_ families for DRTs. The population exhibits wide diversity in pod morphology (length, shape, and color) as well as in seed size and seed coat color.

While the population mean leaned toward the phenotypic value of the recurrent Sam parent, there was a remarkable breadth of variation observed for each trait, highlighting the diversity within the population (Supplementary Fig. 1, Fig. 2). The most notable variation was observed in the length of the main stem, ranging from 15.25 to 239.8 cm, and in the dry haulm weight, which spanned from 20 to 373 g. The minimum and maximum values of the families represent approximately 3 to 4 times the mean value of the recurrent parent. Another significant observation is the variation in flowering and pod maturity times. In 2023, for example, 18% of the genotypes flowered, and 39% matured 50 d later than the recurrent parent.

The values of broad-sense heritability were high for all traits in the 2 yr of experimentation, ranging between 52% and 98%, except for the dry haulm weight and the main stem length in 2023 (Table 2, Supplementary Tables 1 and 2).

### Phenotypic correlations

**Fig. 3.**
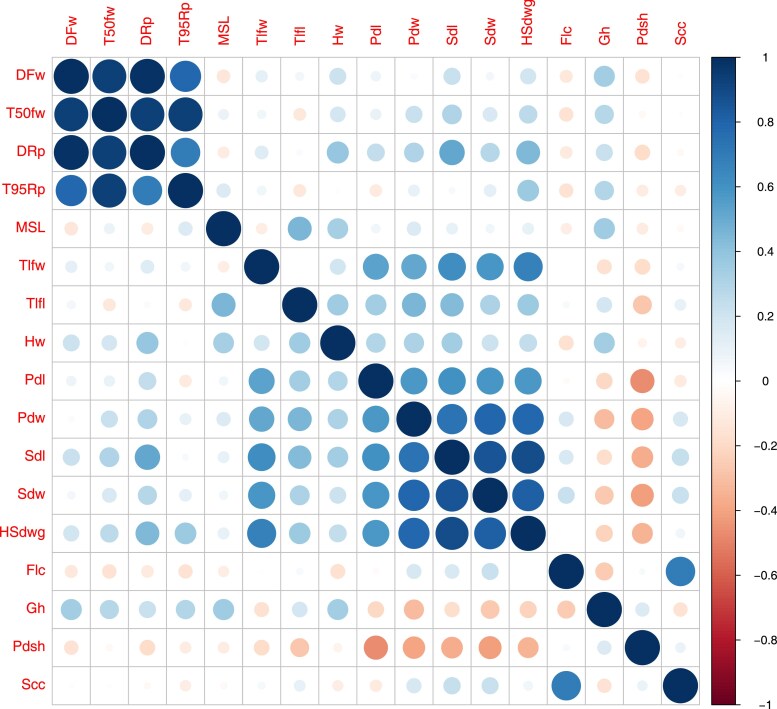
Phenotypic correlation matrix among the measured traits. The strength and direction of pairwise correlations are indicated by the color scale, ranging from −1 (strong negative correlation) to +1 (strong positive correlation). Traits related to flowering time (DFw, T50fw, T95Rp) showed strong positive associations with each other, while reproductive and seed traits (Pdl, Pdw, Sdl, Sdw, HSdwg) were also positively correlated. Some traits, such as Gh and Scc, exhibited weak or no correlation with most other variables.

The phenotypic correlation analysis revealed clear trait groupings. Flowering and maturity traits (DFw, T50fw, T95Rp) were highly positively correlated, reflecting their shared developmental basis. Similarly, pod and seed dimensions (Pdl, Pdw, Sdl, Sdw, HSdwg) clustered together, consistent with coordinated effects on seed size (Fig. 3). Leaf size traits (Tlfl and Tlfw) showed positive correlations with pod and seed dimensions, suggesting that larger vegetative organs may contribute to increased reproductive organ size. Plant height (MSL) was positively associated with flowering time and leaf size traits. By contrast, plant growth habit (Gh) and pod shattering (Pdsh) showed low or negative correlations with yield- or phenology-related traits, suggesting largely independent genetic control (Fig. 3).

### Genetic map

Out of the 2,602 SNPs in the mid-density panel, 1,046 (40.19%) high-quality polymorphic SNPs were utilized to construct the genetic map. This linkage map consists of 11 linkage groups, corresponding to the 11 chromosomes of cowpea. The total length of the map is 1,131.6 cM (Table 3), with chromosome lengths ranging from 40.6 cM (Vu04) to 189.9 cM (Vu03). The number of markers per chromosome ranged from 73 to 132 in chromosomes 2 and 3, respectively. The average distance between markers is highest for chromosome 1 at 1.6 cM, while chromosomes 4 and 8 had the lowest average distance at 0.5 cM.

**Table 3.** Summary statistics of the 11 linkage groups for the SNP map based on the BC1F2 *V. unguiculata* population.

Linkage group	Number of markers	Length (cM)	Average interval (cM)	Maximum interval (cM)
Vu01	80	129.5	1.6	13.9
Vu02	73	75.4	1	15.1
Vu03	132	189.9	1.4	12
Vu04	89	40.6	0.5	6.1
Vu05	97	74.5	0.8	10.2
Vu06	78	116.1	1.5	19.7
Vu07	121	138	1.1	11.7
Vu08	95	50.8	0.5	3
Vu09	114	98.8	0.9	7.3
Vu10	82	99.2	1.2	9.2
Vu11	85	118.8	1.4	12
Total	1,046	1,131.6	1.1	19.7

### Identification of QTLs for DRTs

**Fig. 4.**
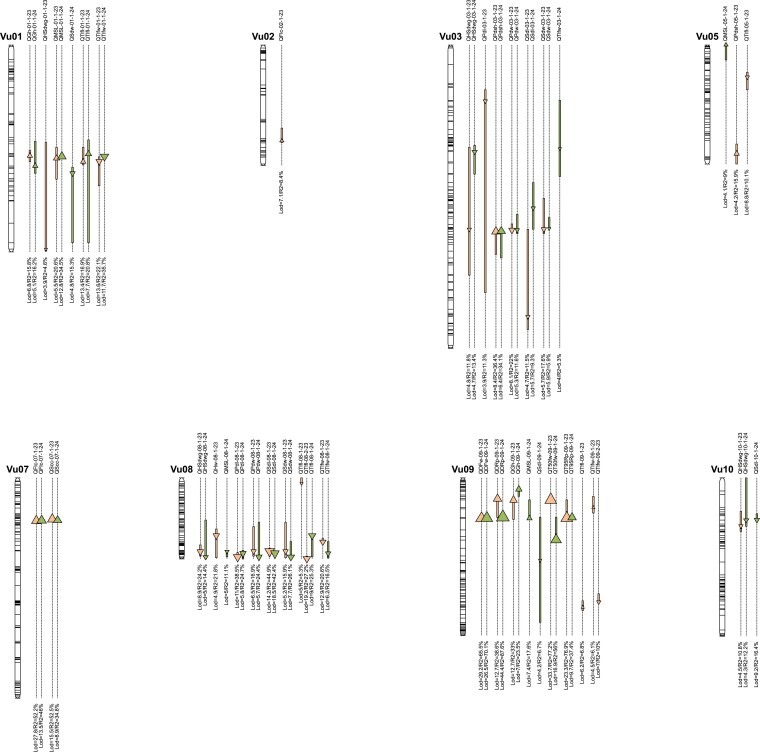
Genetic map of detected QTLs. QTLs are named as “Q” followed by the trait code, chromosome number, and year of detection. Each QTL is shown as a triangle positioned at the peak marker and a box indicating its confidence interval. Triangle orientation reflects the sign of the additive effect (upward = positive effect from the wild parent; downward = positive effect from the cultivated parent), while triangle size is proportional to the proportion of phenotypic variance explained (R^2^). QTLs detected in 2023 are shown in orange, and those detected in 2024 in light green. The LOD score and R^2^ value are reported beneath each QTL.

In this study, we explored the association between phenotype and genotype for 17 DRTs over 2 yr, resulting in the identification of a total of 65 QTLs. The LOD thresholds for declaring a QTL ranged from 3.91 for the pod length QTL (*QPdl-03-1-23*) on chromosome Vu03, to 44.44 for time to first mature pod QTL (*QDRp-09-1-24*) on chromosome Vu09 (Table 4 and Fig. 4). The percentage of phenotypic variation explained by the QTLs varied significantly, from 4.63% for terminal leaf length QTL (*QTlfl-08-1-23*) on chromosome Vu08 to 87.6% for time to first mature pod (*QDRp-09-1-24*) on chromosome Vu09.

**Table 4.** Detected QTLs.

Trait	Chr.	2023										2024									
		Method	QTL	SNP	Peak (cM)	Start (cM)	End (cM)	LOD	R^2^	R^2^tot	Add	Method	QTL	SNP	Peak (cM)	Start (cM)	End (cM)	LOD	R^2^	R^2^tot	Add
Flc	Vu02	MIM and SIM	QFlc-02-1-23	2_04643	60	52	61	7.06	8.44	73.64	0.47										
Flc	Vu07	MIM and SIM	QFlc-07-1-23	2_06783	27	27	28	27.75	52.22	73.64	0.51	MIM and SIM	QFlc-07-1-24	2_06783	27	27	28	13.5	45.97	45.97	0.42
Gh	Vu01	MIM only	QGh-01-1-23	2_27388	69	66	73	6.84	15.81	44.32	0.37	MIM only	QGh-01-1-24	2_30503	76	60	80	5.07	16.2	37.69	0.36
Gh	Vu09	MIM and SIM	QGh-09-1-23	2_18645	14	14	26	12.72	32.95	44.32	0.51	MIM and SIM	QGh-09-1-24	2_23075	7	7	12	7.03	23.54	37.69	0.42
Pdsh	Vu03	MIM and SIM	QPdsh-03-1-23	2_22306	117	115	131	8.35	36.41	51.19	0.22	MIM only	QPdsh-03-1-24	2_22306	117	115	133	6.35	34.15	34.15	0.26
Pdsh	Vu05	MIM only	QPdsh-05-1-23	2_15792	68	62	75	4.22	15.87	51.19	0.28										
Scc	Vu07	MIM and SIM	QScc-07-1-23	2_06783	27	27	27	28.03	73.94	73.94	−0.507	MIM and SIM	QScc-07-1-24	2_06783	27	15	103	5.993	24.99	24.99	−0.295
Tlfl	Vu01	MIM and SIM	QTlfl-01-1-23	2_30503	73	64	75	13.43	16.87	73.37	0.7	MIM and SIM	QTlfl-01-1-24	2_28826	68	59	124	7.68	20.84	50.28	0.58
Tlfl	Vu05	MIM only	QTlfl-05-1-23	2_04365	20	17	28	8.83	10.14	73.37	−0.75										
Tlfl	Vu08	MIM only	QTlfl-08-2-23	2_30357	4	0	4	19.25	27.18	73.37	−1.02	MIM and SIM	QTlfl-08-1-24	2_10921	36	36	49	9.02	25.29	50.28	−0.7
Tlfl	Vu08	MIM and SIM	QTlfl-08-1-23	2_33981	51	49	51	4.95	5.28	73.37	−0.43										
Tlfl	Vu09	MIM only	QTlfl-09-1-23	2_01760	81	77	83	6.22	6.8	73.37	0.42										
Tlfw	Vu01	MIM and SIM	QTlfw-01-1-23	2_30503	73	70	88	13.64	22.11	66.15	−0.65	MIM and SIM	QTlfw-01-1-24	2_33402	70	69	70	11.68	28.91	53.91	−0.7
Tlfw	Vu03											SIM only	QTlfw-03-1-24	2_51359	65	34.56	82.32	4.04	5.26	53.91	−0.04
Tlfw	Vu08	MIM and SIM	QTlfw-08-1-23	2_10291	40	38	41	12.88	20.55	66.15	−0.5	MIM only	QTlfw-08-1-24	2_52149	47	40	50	6.18	11.56	53.91	−0.5
Tlfw	Vu09	MIM only	QTlfw-09-1-23	2_41476	19	12	22	4.48	6.07	66.15	0.23										
Tlfw	Vu09	MIM and SIM	QTlfw-09-2-23	2_01781	78	73	78	7.03	10.01	66.15	−0.3										
MSL	Vu01	MIM and SIM	QMSL-01-1-23	2_33402	71	64	84	5.55	20.55	20.55	23.14	MIM and SIM	QMSL-01-1-24	2_33402	70	68	70	12.8	34.49	56.49	37.7
MSL	Vu05											MIM only	QMSL-05-1-24	2_24585	0	0	9	4.13	9.02	56.49	23.09
MSL	Vu08											MIM only	QMSL-08-1-24	2_19341	46	45	49	4.99	11.1	56.49	−24.87
MSL	Vu09											MIM only	QMSL-09-1-24	2_25344	25	14	25	7.44	17.58	56.49	54.35
DFw	Vu09	MIM and SIM	QDFw-09-1-23	2_25344	25	25	26	29.15	65.55	65.55	17.63	MIM and SIM	QDFw-09-1-24	2_25344	25	25	25	26.47	70.09	70.09	23.09
T50fw	Vu09	MIM and SIM	QT50fw-09-1-23	2_18645	14	12	14	33.72	77.21	77.21	26.51	MIM and SIM	QT50fw-09-1-24	2_18607	39	25	39	16.94	56	56	18.64
DRp	Vu09	MIM and SIM	QDRp-09-1-23	2_44371	13	13	25	12.66	38.58	38.58	30.06	MIM and SIM	QDRp-09-1-24	2_43409	25	25	25	44.44	87.61	87.61	24.04
T95Rp	Vu09	MIM and SIM	QT95Rp-09-1-23	2_25344	25	14	26	23.25	76.94	76.94	22.95	MIM and SIM	QT95Rp-09-1-24	2_43409	25	25	25	9.65	37.36	37.36	25.4
Pdl	Vu03	MIM and SIM	QPdl-03-1-23	2_51815	35	28	155	3.91	11.34	46.28	−9.48										
Pdl	Vu08	MIM and SIM	QPdl-08-1-23	2_25158	49	47	51	11.02	38.46	46.28	−21.23	MIM and SIM	QPdl-08-1-24	2_52149	47	45	51	5.85	24.68	24.68	−14.99
Pdw	Vu03	MIM and SIM	QPdw-03-1-23	2_22313	116	112	117	8.05	22.02	57.38	−0.08	SIM only	QPdw-03-1-24	2_22306	116	106	118	5.34	11.64	36.03	−0.48
Pdw	Vu08	MIM and SIM	QPdw-08-1-23	2_19341	46	30	49	6.45	16.9	57.38	−0.04	MIM and SIM	QPdw-08-1-24	2_12563	49	28	51	5.71	13.88	36.03	−0.61
Sdl	Vu03	MIM and SIM	QSdl-03-1-23	2_30056	170	115	178	4.67	11.48	54.77	−0.4	MIM and SIM	QSdl-03-1-24	2_54847	102	86	115	5.67	9.27	70.71	−0.52
Sdl	Vu08	MIM and SIM	QSdl-08-1-23	2_19341	46	44	49	14.23	44.95	54.77	−1.09	MIM and SIM	QSdl-08-1-24	2_52149	47	46	48	18.48	42.44	70.71	−0.97
Sdl	Vu09											MIM only	QSdl-09-1-24	2_25683	52	25	90	4.24	6.69	70.71	−0.4
Sdl	Vu10											MIM only	QSdl-10-1-24	2_40097	26	22	26	9.16	16.37	70.71	−0.57
Sdw	Vu01											MIM only	QSdw-01-1-24	2_27019	80	77	124	4.82	12.36	49.13	−0.19
Sdw	Vu03	MIM and SIM	QSdw-03-1-23	2_22313	115	96	118	5.7	17.58	44.08	−0.34	SIM only	QSdw-03-1-24	2_22313	115	108	115.2	5.89	5.88	49.13	−0.22
Sdw	Vu08	MIM and SIM	QSdw-08-1-23	2_19341	46	28	50	5.21	15.87	44.08	−0.27	MIM and SIM	QSdw-08-1-24	2_02644	49	40	50	7.66	15.72	49.13	−0.3
Hw	Vu08	MIM and SIM	QHw-08-1-23	2_10921	36	32	50	4.92	21.82	21.82	−5.32										
HSdwg	Vu01	SIM only	QHSdwg-01-1-23	2_30660	128	61	129	3.92	4.63	62.8	−0.295										
HSdwg	Vu03	MIM & SIM	QHSdwg-03-1-23	2_22313	115	64	144	4.85	7.15	62.8	−2.16	MIM and SIM	QHSdwg-03-1-24	2_51359	67	63	81	4.66	13.41	47.1	−1.56
HSdwg	Vu08	MIM and SIM	QHSdwg-08-1-23	2_19341	46	42	49	8.93	22.73	62.8	−2.67	MIM and SIM	QHSdwg-08-1-24	2_02644	49	26	51	4.95	14.36	47.1	−1.78
HSdwg	Vu10	MIM only	QHSdwg-10-1-23	2_08810	30	21	34	4.49	9.4	62.8	−1.85	MIM only	QHSdwg-10-1-24	2_40097	26	0	30	4.3	12.25	47.1	−1.58

QTL name is designated by “Q”, followed by the trait code, the chromosome number, the year. Chr., chromosome number; SNP, closest SNP; Peak, QTL position; Start and End, upper and lower limits of confidence interval; R^2^, percentage of phenotypic variation explained by each QTL; R2tot, percentage of phenotypic variation explained by all QTLs; Add, additive effect (positive value indicates alleles of the wild parent increase trait values).

The number of QTLs detected for each trait ranged from 1 to a maximum of 7. These QTLs were distributed on 8 out of the 11 cowpea chromosomes (Table 4 and Fig. 4). Notably, 78.4% (51) of the QTLs were clustered on 4 chromosomes: Vu01, Vu03, Vu08, and Vu09 (Table 4 and Fig. 4). The QTL clusters include plant growth habit, seed size, main stem length and terminal leaflet size on chromosome Vu01; pod size, seed size, and pod shattering on chromosome Vu03; terminal leaflet size, pod size, and seed size on chromosome Vu08; and time to flowering and pod maturity on chromosome Vu09.

#### Morphology

##### Flower color

Three QTLs were associated with flower color trait in 2023 and 2024. The QTL *QFlc-02-1-23* detected only in 2023 on chromosome Vu02, is a minor QTL accounting for 8.44% of the phenotypic variation. *QFlc-07-1-*23 and QFlc*-07-1-24* were detected in 2023 and 2024 in the same region on chromosome Vu07. They are major QTLs explaining 52.22% and 45.97% of the phenotypic variance, respectively (Table 4 and Fig. 4).

##### Growth habit

Two QTLs, *QGh-09-1-23* and *QGh-09-1-24*, associated with plant growth habit, were identified on chromosome Vu09, 1 in 2023 and the other in 2024. These QTLs explained 32.95% and 23.54% of the phenotypic variation, respectively. Two other QTLs, *QGh-01-1-23* and *QGh-01-1-24,* were detected on chromosome Vu01 for the same trait in 2023 and 2024 explaining 15.81% and 16.20% of the phenotypic variation, respectively. For all detected QTLs the allele from the cultivated parent was associated to an erect growth habit (Table 4 and Fig. 4).

##### Pod shattering

Two major QTLs, *QPdsh-03-1-23 and QPdsh-03-1-24*, explaining 36.41% and 34.15% of the phenotypic variation, respectively, were identified on chromosome Vu03 in 2023 and 2024. Another QTL was detected in 2023 only on chromosome Vu05 (*QPdsh-05-1-23*), explaining 15.87% of the phenotypic variation. The allele of the cultivated parent was associated to resistance to pod shattering for all detected QTLs (Table 4 and Fig. 4).

##### Seed coat color

Two major QTLs, *QScc-07-1-23* and *QScc-07-1-24*, were consistently identified in 2023 and 2024, in the same genomic region on chromosome Vu07. These QTLs explained 73.9% and 25% of the phenotypic variation, respectively (Table 4 and Fig. 4).

##### Terminal leaflet length and width

A total of 14 QTLs were identified for traits related to terminal leaflet characteristics. Specifically, 8 QTLs associated with terminal leaflet length and terminal leaflet width were consistently detected over the 2 yr on chromosome Vu01 (*QTlfl-01-1-23, QTlfl-01-1-24, QTlfw-01-1-23,* and *QTlfw-01-1-24*) and on chromosome Vu08 (*QTlfl-08-2-23, QTlfl-08-1-24, QTlfw-08-1-23*, and *QTlfw-08-1-24*). Each of these QTLs explained 16% to 27% of the phenotypic variation and were considered as major QTLs.

Additionally, 3 QTLs related to terminal leaflet length and 3 QTLs related to terminal leaflet width were identified (*QTlfw-03-1-24, QTlfl-05-1-23, QTlfl-08-1-23, QTlfl-09-1-23, QTlfw-09-1-23, QTlfw-09-2-23*) on chromosomes Vu03, Vu05, Vu08, and Vu09. They explain 5.28% to 10.14% of the phenotypic variation.

Four of the QTLs detected for terminal leaflet traits showed a positive effect attributed to the wild parent: the 2 major QTLs detected for Tlfl in 2023 and 2024 on chromosome Vu01 (QTlfl-01-1-23 and QTlfl-01-1-24) and the 2 minor QTLs detected for Tlfl and Tlfw on Vu09 in 2023 (QTlfl-09-1-23, QTlfw-09-1-23). In contrast, for all other QTLs, the alleles of the cultivated parent contributed positively to the trait variation (Table 4 and Fig. 4). Interestingly, the major QTLs detected for Tlfw on Vu01 and the colocalizing QTLs for Tlfl had opposite effects, indicating that in this region, the wild allele contributed to longer and thinner terminal leaves.

##### Main stem length

Two QTLs (*QMSL-01-1-23* and *QMSL-01-1-24*) were consistently identified in 2023 and 2024 on chromosome Vu01. These QTLs were located in the same region and explained 20.55% and 34.49% of the phenotypic variation, respectively. The alleles from the wild parent were associated with an increase in the main stem length (Table 4 and Fig. 4). In addition, 3 other QTLs were detected in 2024 on chromosomes Vu05, Vu08 and Vu09 (*QMSL-05-1-24, QMSL-08-1-24, QMSL-09-1-24*), explaining 9.02%, 11.10%, and 17.58% of the phenotypic variation, respectively. The QTL *QMSL-08-1-24* was the only QTL for the length of the main stem that had a positive effect contributed by the cultivated parent.

#### Phenology

##### Time to 1st flower and time to 50% flowering

Two major QTLs were consistently identified in 2023 and 2024 for each trait: *QDFw-09-1-23* and *QDFw-09-1-24* for the time to first flower, and *QT50fw-09-1-23* and *QT50fw-09-1-*24 for the time to 50% flowering. These QTLs are located in the same region on chromosome Vu09 and account for 56% to 77% of the phenotypic variation observed. At these QTLs, the alleles of the cultivated parent were associated with early flowering (Table 4 and Fig. 4).

##### Time to 1st mature pod and time to 95% pod maturity

Two major QTLs were consistently identified in 2023 and 2024 for each trait: *QDRp-09-1-23* and *QDRp-09-1-24* for the time to first mature pod, and *QT95Rp-09-1-23* and *QT95Rp-09-1-24* for the time to 95% pod maturity. The 4 QTLs are located in the same region on chromosome Vu09 and account for 37.4% to 87.6% of the phenotypic variation observed. At the 4 QTLs, the alleles of the cultivated parent were associated with early maturing (Table 4 and Fig. 4).

#### Yield components

##### Pod length, pod width, seed length, and seed width

A major cluster of 8 QTLs, identified in 2023 and 2024 for pod length, pod width, seed length, and seed width, was located at the end of chromosome Vu08 (*QPdl-08-1-23, QPdl-08-1-24, QPdw-08-1-23* consistently*, QPdw-08-1-24, QSdl-08-1-23, QSdl-08-1-24, QSdw-08-1-23*, and *QSdw-08-1-24*). These QTLs explained from 15.9% to 44.9% of the phenotypic variation.

Another major cluster of 7 QTLs, was also found on chromosome Vu03, with 4 QTLs detected in 2023 for the 4 traits (*QPdl-03-1-23, QPdw-03-1-23, QSdl-03-1-23*, and *QSdw-03-1-23*) explaining from 11.34% to 22.02% of the phenotypic variation and 3 QTLs for seed length, and pod and seed width detected in 2024 (*QSdl-03-1-24, QPdw-03-1-24, QSdw-03-1-24*) explaining 5.88% to 11.64% of the phenotypic variation.

In addition, 3 minor QTLs were detected in 2024, 2 for seed length on chromosome Vu09 and Vu10 (*QSdl-09-1-24* and *QSdl-10-1-24*) and 1 for seed with on chromosome Vu01 (*QSdw-01-1-24*), accounting for 6.69%, 16.37%, and 15.30% of the phenotypic variation, respectively (Table 4 and Fig. 4).

Remarkably, the allele of the cultivated parent was associated with increases in pod and seed size at all detected QTLs.

##### Dry haulm weight

One major QTL, *QHw-08-1-23*, explaining 21.82% of the phenotypic variation was identified on chromosome Vu08 in 2023. The allele of the cultivated parent is associated with the increase of the dry above-ground biomass (Table 4 and Fig. 4).

##### One hundred seed weight

Six QTLs (*QHSdwg-03-1-23, QHSdwg-03-1-24, QHSdwg-08-1-23, QHSdwg-08-1-24, QHSdwg-10-1-*23, and QHSdwg*-10-1-24*) were consistently identified for 100-seed weight in 2023 and 2024 on chromosomes Vu03, Vu08, and Vu10, accounting for 10.8% to 24.2% of the phenotypic variation. One additional QTL (*QHSdwg-01-1-23*) was identified in 2023 on chromosome Vu01. The QTLs located on chromosome Vu03 and Vu08, were in the same region as those for pod width, seed length and seed width, while the QTL located on chromosome Vu10 was associated to a seed length QTL detected in 2024, and the QTL located on chromosome Vu01 was in the same region as those for seed width, plant growth habit, main stem length and leaf size. Similarly to pod width, seed length and seed width, the alleles from the cultivated parent at the QTL loci are associated with increased 100-seed weight (Table 4 and Fig. 4).

### QTLs comparison

In our study, we compared the identified QTLs with those reported by Lo et al. (2018) and Andargie et al. (2014). Since both our study and that of Lo et al. (2018) used SNP markers, we were able to directly compare the genomic regions associated with 7 common DRTs. Lo et al. (2018) identified 2 QTLs for pod shattering: 1 located on chromosome Vu03 and the other on chromosome Vu05, which correspond to the QTL regions we identified in this study (Table 5). Furthermore, we identified overlapping QTL regions for several traits: flower color on chromosome Vu07, days to flowering on chromosome Vu09, 100-seed weight on chromosomes Vu01 and Vu08, pod length on chromosomes Vu03 and Vu08, and seed length and width on chromosomes Vu08 and Vu01 (Table 4). Finally, 1 QTL for 100-seeds weight on chromosome Vu06 was specific to the study by Lo et al. (2018), while 2 QTLs for 100-seed weight located on chromosomes Vu03 and Vu10 were unique to our study (data not shown).

**Table 5.** QTL comparison between our study, Andargie et al. (2014), and Lo et al. (2018).

Traits	Andargie et al. (2014)				Lo et al. (2018)				Current study			
	Chr.	SSR (QTL peak)	Physical pos. (bp)	R^2^ (%)	Chr.	SNP (QTL peak)	Physical pos. (bp)	R^2^ (%)	Chr.	SNP (QTL peak)	Physical pos (bp)	R^2^ (%)
Pod shattering	Vu03	SSR-6733	48,979,553	17.2	Vu03	2_08497	41,972,285	37.6	Vu03	2_22306	39,436,737	36.41
Pod shattering	Vu05	SSR-6369	33,060,537	6.4	Vu05	2_23044	38,510,454	30.2	Vu05	2_15792	39,019,423	15.87
Pod shattering	Vu09	SSR-7008	5,723,632	16.6								
Pod shattering	Vu11	SSR-6663	41,522,204	7.7								
Flower color	Vu09	SSR-6701	29,890,723	-	Vu07	2_24259	40,615,468	85.6	Vu07	2_06783	41,471,771	50.64
Days to flowering					Vu09	2_03945	30,938,517	79.3	Vu09	2_25344	29,911,522	70.09
100-seeds weight	Vu01	SSR-6919	38,077,223	9.2	Vu01	2_17042	46,381,535	19.8	Vu01	2_30660	41,245,116	3.92
100-seeds weight	Vu02	SSR-6924	25,161,626	13.3								
100-seeds weight	Vu05	SSR-6314	47,479,861	8.9								
100-seeds weight					Vu08	2_05809	58,735,955	36.8	Vu08	2_19341	58,003,268	24.2
100-seeds weight	Vu09	SSR-6701	29,890,723	10.1								
100-seeds weight	Vu09	SSR-6705	42,984,594	13.8								
Pod length					Vu08	2_00195	58,388,599	46	Vu08	2_25158	59,114,638	38.46
Pod length					Vu03	2_49508	54,283,830	23.9	Vu03	2_51815	43,340,178	11.34
Leaf length					Vu08	2_09764	58,849,776	38.2	Vu08	2_30357	59,462,190	27.18
Leaf width					Vu01	2_19941	44,531,359	63.2	Vu01	2_30503	45,471,000	22.11
Leaf width					Vu08	2_21200	58,786,169	34.7	Vu08	2_10291	53,155,693	20.55

To compare the QTL regions identified for 3 traits reported by Andargie et al. (2014), we first performed a BLAST search of the SSR sequences within the cowpea genome to determine the physical location of the SSR at each QTL peak. We found a high degree of consistency in the QTL regions for pod shattering across all 3 studies. Notably, the pod-shattering QTL on chromosome Vu03 was identified in all 3 studies, while the QTL on chromosome Vu05 was consistent with the findings of Lo et al. (2018) (Table 5). Additionally, 2 other QTLs reported by Andargie et al. (2014) were unique to this study: 1 with a major effect located on chromosome Vu09 and another with a minor effect on chromosome Vu11 (Table 5). In contrast, for the trait of 100-seed weight, there was only 1 common region shared among the 3 studies, located on chromosome Vu01. Four other QTLs were specific to Andargie et al. (2014). This included 3 QTLs with major effects—2 located on chromosome Vu09 and 1 on chromosome Vu02—as well as 1 QTL with a minor effect on chromosome Vu05. Likewise, the C locus responsible for both flower and seed coat color was mapped on chromosome Vu09 (Table 5).

## Discussion

In this study, we developed an interspecific backcross population by crossing the elite variety Sam with a wild accession of *V. unguiculata* var. *spontanea* collected from Senegal. The population was evaluated over 2 consecutive years, during which 65 QTLs associated with 17 DRTs were identified, with 55 QTLs explaining more than 10% of the phenotypic variance. It is important to note that the population sizes used for QTL detection (126 individuals in 2023 and 101 in 2024) were relatively limited. Small population sizes can reduce the precision of QTL effect estimation and increase the likelihood of overestimating their magnitude, a phenomenon known as the Beavis effect (Xu 2003).

Among the 65 QTLs, 48 QTLs (73.8%) were consistently detected in both 2023 and 2024. These QTLs include those associated with main stem length on chromosome Vu01, terminal leaf length and width on chromosomes Vu01 and Vu08, pod and seed length and width, 100-seed weight on chromosomes Vu03, Vu08, and Vu10; flower and seed coat color on chromosome Vu07; flowering time; maturity time; and plant growth habit on chromosome Vu09 (Fig. 4). Since the QTLs affecting the same trait were consistently identified in similar locations on the same chromosomes, and all exhibit major effects, accounting for more than 10% of the genetic variation, it is reasonable to consider them as the same QTLs (Paterson 2002). Based on this assumption, the total number of distinct QTLs detected in this study can be reduced to 41, with an average of 2.4 QTL identified per trait. These findings align well with previous reports on the number of genes/QTLs controlling DRTs in legume crops (Ambika et al. 2022; Verma et al. 2022).

### QTL clustering particular feature of domestication

In crop domestication, only a few regions have been identified as significantly altering the key traits that distinguish cultivated species from their wild ancestors (Gepts 2004; Ross-Ibarra 2005; Geleta and Ortiz 2016). Our study provides compelling evidence of such clustering of domestication-related QTLs in cowpea. We identified 4 major genomic regions, on chromosomes Vu01, Vu03, Vu08, and Vu09, that collectively harbor most of the QTLs for DRTs. Loci associated with changes in organ size such as leaf, pod, and seed dimensions were mainly concentrated on Vu01, Vu03, and Vu08, whereas loci influencing phenology, such as flowering time and pod maturity, were localized on Vu09. These QTL patterns are consistent with the high phenotypic correlations we observed among organ size traits, and among phenology traits. Changes in organ size, commonly referred to as gigantism, are a key characteristic of crop domestication (Weeden 2007). Clustering of QTLs associated with gigantism-related traits has been reported in zombi pea (*Vigna vexillata*) for leaf width and seed thickness (Dachapak et al. 2018), and for seed size and stem thickness (Amkul et al. 2020). Similar clustering has also been observed in Azuki bean (*Vigna angularis*), yardlong bean (*V. unguiculata L.* Walp. ssp. *sesquipedalis*) for stem thickness, leaf width, and seed size (Kaga et al. 2008; Kongjaimun et al. 2012), and in grain cowpea for leaf width and seed size (Lo et al. 2018). Interestingly, in our study the QTL responsible for pod shattering was aligned with the seed size QTLs on Vu03, while the QTL influencing plant growth habit co-located with flowering and pod maturity loci on chromosome Vu09, while these traits showed no phenotypic correlations. These findings suggest that either genes with pleiotropic effect or tightly linked gene clusters governing various DRTs, which provide a strong selective advantage, may have been selected during cowpea domestication and subsequent improvement on this crop (Paterson 2002; Sweeney and McCouch 2007; Qian et al. 2016). This likely led to the rapid development of morphotypes with shorter growth duration, reduced pod shattering, larger seed size, and modified growth habits during cowpea domestication, making them better suited for cultivation.

### Different loci may have been selected during the 2 independent domestication events of cowpea

The question of whether cowpea underwent single or double domestication events has long been a topic of debate. Using AFLP and RAPD markers, Coulibaly et al. (2002) and Ba et al. (2004) each argued in favor of a single domestication event in West Africa. The West African origin of cowpea was further supported by archaeological evidence from central Ghana, which shows that cowpea was cultivated in the region between 1830 and 1595 Bce (D’Andrea et al. 2007). More recently, Fiscus et al. (2024) examined a large collection of cowpea germplasm from IITA using a 50k SNP array. Their findings confirmed the West African origin of domesticated cowpea, based on the observation of the largest number of segregating SNP sites in this region. In contrast, Xiong et al. (2018) suggested a single domestication event in East Africa, based on genetic diversity analysis of 768 accessions from a global germplasm collection, characterized using 11 phenotypic traits and 1,048 SNP markers. Finally, Huynh et al. (2013) and Xiong et al. (2016) conducted comprehensive analyses of the population structure of cultivated and wild cowpeas using SNP markers. Their research demonstrates that cultivated cowpeas in both East and West Africa are directly linked to local wild cowpea species, suggesting 2 independent domestication regions. This hypothesis was further supported by linguistic data presented by Herniter et al. (2020).

We compared the QTL regions identified in this study with those reported by Andargie et al. (2014) and Lo et al. (2018). We observed striking QTL positional consistency, with notably more shared QTL between Lo et al. (2018) and our study than between either of these and Andargie et al. (2014). Two QTL regions on chromosomes Vu03 and Vu05, both associated with pod shattering, were shared across all 3 studies. This was unexpected, given that Lo et al. (2018) reported no overlapping QTL with Andargie et al. (2014) in their own comparison. Notably, 2 pod-shattering QTLs on chromosome Vu09 and Vu11 was unique to Andargie et al. (2014). For flower color, we identified a QTL on chromosome Vu07 in common with Lo et al. (2018), whereas Andargie et al. (2014) mapped this trait to chromosome Vu09. For 100-seed weight, Andargie et al. (2014) detected 5 QTLs, while Lo et al. (2018) and our study identified 3 and 4 regions, respectively. The QTL on chromosome Vu01 was shared across all 3 studies, while 1 QTL on chromosome Vu08 were common between our study and Lo et al. (2018). Seed size is widely recognized as a hallmark of the domestication syndrome in seed-propagated crops, with strong directional selection during domestication leading to larger seeds (Fuller 2007; Purugganan and Fuller 2009). However, unlike traits controlled by 1 or a few major domestication genes, seed weight in cowpea appears to be quantitatively inherited, influenced by many loci. Furthermore, post-domestication diversification and local adaptation may have targeted different alleles for seed size in distinct breeding pools, reducing the likelihood of repeatedly detecting the same genomic regions across studies.

Finally, we found overlapping QTL with Lo et al. for flowering time (Vu09) and leaf size–related traits (Vu03 and Vu08). Overall, these results indicate that while certain domestication-related QTLs, such as those for pod shattering, are conserved across populations and environments, others diverge.

To further deepen our understanding of cowpea domestication, we examined the genetic pools used to develop the populations in the different studies. Notably, the wild species used in Andargie et al. (2014) originated from coastal Kenya, while the cultivated parent, 524B, was derived from a cross between the California cultivars CB5 and CB3. Recent research by Fiscus et al. (2024) revealed that 71% of the genetic material used in the U.S. cowpea breeding program, including CB5, can be traced back to the East African genepool. These findings suggest that the population developed by Andargie et al. (2014) was predominantly derived from the East African cowpea genepool. Interestingly, the wild and the cultivated species in Lo et al. (2018) were from Central and West Africa, respectively, while our study focused on species from Senegal (West Africa). Assuming that the wild and cultivated species in Lo et al. (2018) and in this study belong to the same genepool (Fiscus et al. 2024) and are distinct from those in East Africa (as outlined by Huynh et al. 2013; Xiong et al. 2016, Fiscus et al. 2024), we can hypothesize that the differences in QTL regions are attributable to the varying regions of origin of the species used. The divergence in QTL regions across the studies may support a dual domestication scenario. We hypothesize that both common and distinct loci may have been favored during the separate domestication events of cowpea in East and West Africa. This pattern mirrors the dual domestication of common beans, where both unique and overlapping regions under selection were identified for Mesoamerican and Andean domestication events (Schmutz et al. 2014). For example, in this legume crop species, pod shattering is associated with 2 QTLs (Pv03 and Pv08) in the Mesoamerican genepool, whereas 4 QTLs (Pv03, Pv05, Pv08, and Pv09) have been identified in the Andean genepool, with Pv03 and Pv08 shared between both (Parker et al. 2021a, 2021b).

Our finding underscores the complex genetic architecture that shapes crop domestication, emphasizing the necessity for further research into the genetic foundations of this human-driven process.

### Wild cowpea species contributed few major QTLs with positive effect to yield-related traits

Wild relatives are an invaluable reservoir of genetic diversity, offering essential traits that can significantly boost crop productivity and adaptability (Tanksley and McCouch 1997; Gur and Zamir 2004). Our findings align with those reported by Lo et al. (2018) suggesting that only a few QTLs showed a positive contribution from wild species. These include traits such as early flowering located on chromosome Vu05 and the number of seeds per pod on chromosome Vu09 (Lo et al. 2018), along with pod length on chromosome Vu09 in this study. These findings contrast with previous studies on cowpea that reported a higher number of QTLs with positive contributions from wild species. For example, in yardlong bean, Kongjaimun et al. (2012) identified 19 out of 97 QTLs for yield-related traits—such as pod size, seed size, flowering precocity, total seed weight, and total number of pods per plant—where wild alleles contributed positively. However, among those 19 QTLs, only 2 had major effects, accounting for 19.6% and 15.6% of the phenotypic variance for the date to flowering and the total number of pods, respectively. In grain cowpea, of the 6 QTLs identified for seed size (100-seed weight trait), wild alleles contributed positively at 3 QTL loci, with only 1 having a major effect that accounted for 13.8% of the phenotypic variation (Andargie et al. 2014). In our study, the limited size of the mapping population reduced its power to detect small-effect QTLs, which may explain the low number of QTLs with positively contributing wild alleles.

Although cowpea wild relatives have limited contributions to yield-related traits they represent major sources of insect pest and disease resistance (Boukar et al. 2020).

## Conclusion

This study sheds light on the genetic architecture underlying DRTs in cowpea. The consistent set of major-effect QTLs identified across 2 yr and their clustering in specific chromosomal regions, confirm that domestication in cowpea, like in other crops, has relied on selection in a few key genomic hotspots. The fact that many of these QTLs co-localize and affect traits such as seed size, pod shattering, and flowering time further suggests that domestication may have favored tightly linked gene complexes that rapidly conferred desirable agronomic traits. Our comparative analysis with previous studies reinforces the hypothesis that cowpea may have undergone at least 2 independent domestication events, as evidenced by both shared and divergent QTL regions among populations from different African regions.

While the wild cowpea parent contributed positively to only a few major traits, these contributions are nonetheless valuable, particularly for traits like pod length, and seed number, and seed size. These findings echo broader trends in crop improvement, where wild relatives often offer unique alleles that are underrepresented in elite germplasm. However, in our study the relatively low number of positive-effect QTLs from the wild parent may reflect limitations in population size and mapping resolution rather than a true absence of useful diversity. Taken together, our results support the continued use of interspecific populations to uncover domestication loci and novel alleles for crop improvement. Future research should focus on fine-mapping the most promising QTLs, exploring their underlying genes, and leveraging genomic tools to integrate wild diversity into breeding programs more effectively.

## Supplementary Material

jkaf248_Supplementary_Data

## Data Availability

All data are available centrally at FigShare (https://doi.org/10.25387/g3.28902872). The files used for the QTL mapping in 2023 (QTLs MAPPING DATA_2023.csv) and 2024 (QTLs MAPPING DATA_2024.csv) are provide as r/qtl data format. The first column is the genotype name, followed by 17 columns for the traits, and then the genotyping data for each genotype (column S to ANX). All trials were conducted in Senegal, ISRA/CNRA of Bambey research station (14.42° N and 16.28° W). Supplemental material available at *G3* online.
