# Supplementary material for: Genetic architecture of cowpea domestication: QTL mapping and comparison shed new light on the dual domestication events
Source: G3 (Bethesda). 2025 Oct 17;16(1):jkaf248. doi: 10.1093/g3journal/jkaf248 (PMC12774598; doi:10.1093/g3journal/jkaf248)
Supplement: jkaf248_Supplementary_Data [file jkaf248_supplementary_data.zip › Supplemental_Material_G3-2025-406133(2).pdf]

```
Response: DFw
            Sum Sq  Df  F value    Pr(>F)
(Intercept)   2534   1 995.5090 < 2.2e-16 ***
Pedigree     41074 125 129.0792 < 2.2e-16 ***
Rep             16   2   3.1917  0.043115 *
Rep:Bloc       178  33   2.1157  0.000834 ***
Residuals      532 209
---
Signif. codes:  0 ‘***’ 0.001 ‘**’ 0.01 ‘*’ 0.05 ‘.’ 0.1 ‘ ’ 1
```

```
Response: T50fw
            Sum Sq  Df   F value  Pr(>F)
(Intercept) 1878.4   1 1076.7474 < 2e-16 ***
Pedigree    4604.2 104   25.3778 < 2e-16 ***
Rep            1.5   2    0.4262 0.65375
Rep:Bloc      92.1  33    1.5997 0.03048 *
Residuals    272.1 156
---
Signif. codes:  0 ‘***’ 0.001 ‘**’ 0.01 ‘*’ 0.05 ‘.’ 0.1 ‘ ’ 1
```

```
Response: DRp
             Sum Sq  Df   F value Pr(>F)
(Intercept)  4675.1   1 2352.9418 <2e-16 ***
Pedigree    17223.5 116   74.7287 <2e-16 ***
Rep             1.7   2    0.4256 0.6541
Rep:Bloc       77.5  33    1.1825 0.2425
Residuals     359.6 181
---
Signif. codes:  0 ‘***’ 0.001 ‘**’ 0.01 ‘*’ 0.05 ‘.’ 0.1 ‘ ’ 1
```

```
Response: T95Rp
            Sum Sq Df  F value  Pr(>F)
(Intercept) 2933.7  1 933.2157 < 2e-16 ***
Pedigree    6560.2 72  28.9832 < 2e-16 ***
Rep           18.4  2   2.9202 0.06176 .
Rep:Bloc     175.9 33   1.6958 0.03827 *
Residuals    185.5 59
---
Signif. codes:  0 ‘***’ 0.001 ‘**’ 0.01 ‘*’ 0.05 ‘.’ 0.1 ‘ ’ 1
```

```
Response: Hw
             Sum Sq Df F value    Pr(>F)
(Intercept)  1115.3  1 28.1578 8.426e-07 ***
Pedigree    13853.6 91  3.8434 5.817e-10 ***
Rep           591.5  2  7.4669  0.001017 **
Rep:Bloc     4716.9 33  3.6086 9.227e-07 ***
Residuals    3446.1 87
---
Signif. codes:  0 ‘***’ 0.001 ‘**’ 0.01 ‘*’ 0.05 ‘.’ 0.1 ‘ ’ 1
```

```
Response: Tlfl
            Sum Sq  Df  F value    Pr(>F)    
(Intercept) 171.95   1 527.4200 < 2.2e-16 ***
Pedigree    378.72 125   9.2929 < 2.2e-16 ***
Rep           0.48   2   0.7417    0.4776    
Rep:Bloc     48.31  33   4.4901 9.412e-12 ***
Residuals    67.49 207                      
---
Signif. codes:  0 ‘***’ 0.001 ‘**’ 0.01 ‘*’ 0.05 ‘.’ 0.1 ‘ ’ 1

Response: Tlfw
             Sum Sq  Df  F value    Pr(>F)    
(Intercept)  20.375   1 119.4945 < 2.2e-16 ***
Pedigree    287.965 124  13.6195 < 2.2e-16 ***
Rep           0.309   2   0.9058    0.4058    
Rep:Bloc     26.790  33   4.7611  9.81e-13 ***
Residuals    36.149 212                      
---
Signif. codes:  0 ‘***’ 0.001 ‘**’ 0.01 ‘*’ 0.05 ‘.’ 0.1 ‘ ’ 1


Response: Pdl
            Sum Sq Df  F value  Pr(>F)    
(Intercept)  85.14  1 111.9598 < 2e-16 ***
Pedigree    696.89 93   9.8537 < 2e-16 ***
Rep           1.49  2   0.9806 0.37930    
Rep:Bloc     41.40 33   1.6495 0.03458 *  
Residuals    64.64 85                    
---
Signif. codes:  0 ‘***’ 0.001 ‘**’ 0.01 ‘*’ 0.05 ‘.’ 0.1 ‘ ’ 1


Response: Pdw
             Sum Sq Df  F value    Pr(>F)    
(Intercept) 0.32085  1 266.8000 < 2.2e-16 ***
Pedigree    1.61766 90  14.9460 < 2.2e-16 ***
Rep         0.03311  2  13.7645 8.465e-06 ***
Rep:Bloc    0.19583 33   4.9345 6.965e-09 ***
Residuals   0.08779 73                      
---
Signif. codes:  0 ‘***’ 0.001 ‘**’ 0.01 ‘*’ 0.05 ‘.’ 0.1 ‘ ’ 1


Response: Sdl
             Sum Sq Df  F value  Pr(>F)    
(Intercept)  35.396  1 275.5677 < 2e-16 ***
Pedigree    213.804 94  17.7076 < 2e-16 ***
Rep           0.050  2   0.1953 0.82295    
Rep:Bloc      7.759 33   1.8306 0.01235 *  
Residuals    12.203 95                    
---
Signif. codes:  0 ‘***’ 0.001 ‘**’ 0.01 ‘*’ 0.05 ‘.’ 0.1 ‘ ’ 1
```

```
Response: Sdw
            Sum Sq Df  F value Pr(>F)
(Intercept) 18.786  1 381.1454 <2e-16 ***
Pedigree    36.354 95   7.7642 <2e-16 ***
Rep          0.057  2   0.5757 0.5642
Rep:Bloc     1.896 33   1.1656 0.2776
Residuals    4.830 98
---
Signif. codes:  0 ‘***’ 0.001 ‘**’ 0.01 ‘*’ 0.05 ‘.’ 0.1 ‘ ’ 1
```

```
Response: MSL

           Df Sum Sq Mean Sq F value    Pr(>F)
Pedigree  110 269990  2454.5  2.7641 1.592e-08 ***
Rep         2   8755  4377.3  4.9295  0.008616 **
Rep:Bloc   32  25824   807.0  0.9088  0.610928
Residuals 132 117214   888.0
```
