# Supplementary material for: Genetic architecture of cowpea domestication: QTL mapping and comparison shed new light on the dual domestication events
Source: G3 (Bethesda). 2025 Oct 17;16(1):jkaf248. doi: 10.1093/g3journal/jkaf248 (PMC12774598; doi:10.1093/g3journal/jkaf248)
Supplement: jkaf248_Supplementary_Data [file jkaf248_supplementary_data.zip › Supplemental_Material_G3-2025-406133(3).pdf]

```
Response: DFw
             Sum Sq  Df  F value Pr(>F)    
(Intercept)  1000.3   1 161.7818 <2e-16 ***
Pedigree    17729.8 100  28.6736 <2e-16 ***
Rep             3.8   2   0.3061 0.7367    
Rep:Bloc      231.8  30   1.2495 0.1913    
Residuals     989.3 160                    
---
Signif. codes:  0 ‘***’ 0.001 ‘**’ 0.01 ‘*’ 0.05 ‘.’ 0.1 ‘ ’ 1

Response: T50fw
             Sum Sq  Df  F value    Pr(>F)    
(Intercept)   817.4   1 125.2550 < 2.2e-16 ***
Pedigree    16439.7  94  26.8007 < 2.2e-16 ***
Rep            15.3   2   1.1759  0.311651    
Rep:Bloc      370.7  30   1.8935  0.007385 ** 
Residuals     887.5 136                       
---
Signif. codes:  0 ‘***’ 0.001 ‘**’ 0.01 ‘*’ 0.05 ‘.’ 0.1 ‘ ’ 1

Response: DRp
            Sum Sq  Df  F value Pr(>F)    
(Intercept) 2170.5   1 527.5149 <2e-16 ***
Pedigree    9747.4  97  24.4221 <2e-16 ***
Rep           14.8   2   1.8041 0.1688    
Rep:Bloc     119.7  30   0.9693 0.5193    
Residuals    522.6 127                    
---
Signif. codes:  0 ‘***’ 0.001 ‘**’ 0.01 ‘*’ 0.05 ‘.’ 0.1 ‘ ’ 1

Response: T95Rp
             Sum Sq  Df  F value    Pr(>F)    
(Intercept)  2443.5   1 769.1066 < 2.2e-16 ***
Pedigree    17984.8  94  60.2202 < 2.2e-16 ***
Rep            41.5   2   6.5238  0.002077 ** 
Rep:Bloc      369.6  30   3.8777 8.542e-08 ***
Residuals     362.2 114                       
---
Signif. codes:  0 ‘***’ 0.001 ‘**’ 0.01 ‘*’ 0.05 ‘.’ 0.1 ‘ ’ 1

Response: Hw
            Sum Sq  Df F value    Pr(>F)    
(Intercept)   5130   1  5.9203  0.016707 *  
Pedigree    410284  91  5.2036 3.603e-15 ***
Rep            910   2  0.5251  0.593069    
Rep:Bloc     58188  30  2.2386  0.001505 ** 
Residuals    88376 102                      
---
Signif. codes:  0 ‘***’ 0.001 ‘**’ 0.01 ‘*’ 0.05 ‘.’ 0.1 ‘ ’ 1


HSdwg
             Df Sum Sq Mean Sq F value Pr(>F)    
Pedigree     94 2018.5  21.473   5.721 <2e-16 ***
```

```
Rep            2   36.1  18.043   4.807 0.0102 *
Rep:Bloc      29  112.8   3.890   1.036 0.4307
Residuals    100  375.3   3.753
---
Signif. codes:  0 ‘***’ 0.001 ‘**’ 0.01 ‘*’ 0.05 ‘.’ 0.1 ‘ ’ 1

Response: Tlfl
             Sum Sq  Df  F value Pr(>F)
(Intercept)  68.244   1 140.5460 <2e-16 ***
Pedigree    251.393 100   5.1774 <2e-16 ***
Rep           1.612   2   1.6601 0.1935
Rep:Bloc     20.253  30   1.3904 0.1017
Residuals    75.748 156
---
Signif. codes:  0 ‘***’ 0.001 ‘**’ 0.01 ‘*’ 0.05 ‘.’ 0.1 ‘ ’ 1

Response: Tlfw
             Sum Sq  Df F value    Pr(>F)
(Intercept)   7.480   1 22.2913 5.041e-06 ***
Pedigree    176.280 100  5.2536 < 2.2e-16 ***
Rep           0.047   2  0.0705    0.9319
Rep:Bloc     33.445  30  3.3225 5.132e-07 ***
Residuals    54.358 162
---
Signif. codes:  0 ‘***’ 0.001 ‘**’ 0.01 ‘*’ 0.05 ‘.’ 0.1 ‘ ’ 1

Response: MSL
            Sum Sq  Df F value    Pr(>F)
(Intercept)  10194   1 15.2908 0.0001389 ***
Pedigree    556795 100  8.3520 < 2.2e-16 ***
Rep           2214   2  1.6602 0.1935476
Rep:Bloc     38676  30  1.9338 0.0052926 **
Residuals   100666 151
---
Signif. codes:  0 ‘***’ 0.001 ‘**’ 0.01 ‘*’ 0.05 ‘.’ 0.1 ‘ ’ 1

Response:Pdl
Df Sum Sq Mean Sq F value   Pr(>F)
Pedigree    94  772.6   8.219   7.299  < 2e-16 ***
Rep          2    0.6   0.313   0.278    0.758
Rep:Bloc    29  103.4   3.566   3.166 2.11e-05 ***
Residuals   84   94.6   1.126
---
Signif. codes:  0 ‘***’ 0.001 ‘**’ 0.01 ‘*’ 0.05 ‘.’ 0.1 ‘ ’ 1

Response:Pdw

Df Sum Sq  Mean Sq F value   Pr(>F)
Pedigree    93 1.2086 0.012995   6.018  < 2e-16 ***
Rep          2 0.0322 0.016125   7.467 0.000978 ***
Rep:Bloc    29 0.0695 0.002397   1.110 0.343893
Residuals   94 0.2030 0.002160
---
Signif. codes:  0 ‘***’ 0.001 ‘**’ 0.01 ‘*’ 0.05 ‘.’ 0.1 ‘ ’ 1
```

```
Repose : Sdl
   Df Sum Sq Mean Sq F value  Pr(>F)
Pedigree     94 196.63  2.0918  11.915 < 2e-16 ***
Rep           2   0.94  0.4709   2.682 0.07314 .
Rep:Bloc     29  10.24  0.3530   2.011 0.00553 **
Residuals   104  18.26  0.1756
---
Signif. codes:  0 ‘***’ 0.001 ‘**’ 0.01 ‘*’ 0.05 ‘.’ 0.1 ‘ ’ 1

Reponse: Sdw
Df Sum Sq Mean Sq F value   Pr(>F)
Pedigree     94 29.846  0.3175   9.831  < 2e-16 ***
Rep           2  1.142  0.5712  17.688 2.44e-07 ***
Rep:Bloc     29  3.069  0.1058   3.277 5.01e-06 ***
Residuals   104  3.359  0.0323
---
Signif. codes:  0 ‘***’ 0.001 ‘**’ 0.01 ‘*’ 0.05 ‘.’ 0.1 ‘ ’ 1
```
