# Supplementary material for: Genetic architecture of cowpea domestication: QTL mapping and comparison shed new light on the dual domestication events
Source: G3 (Bethesda). 2025 Oct 17;16(1):jkaf248. doi: 10.1093/g3journal/jkaf248 (PMC12774598; doi:10.1093/g3journal/jkaf248)
Supplement: jkaf248_Supplementary_Data [file jkaf248_supplementary_data.zip › Supplemental_Material_G3-2025-406133.pdf]

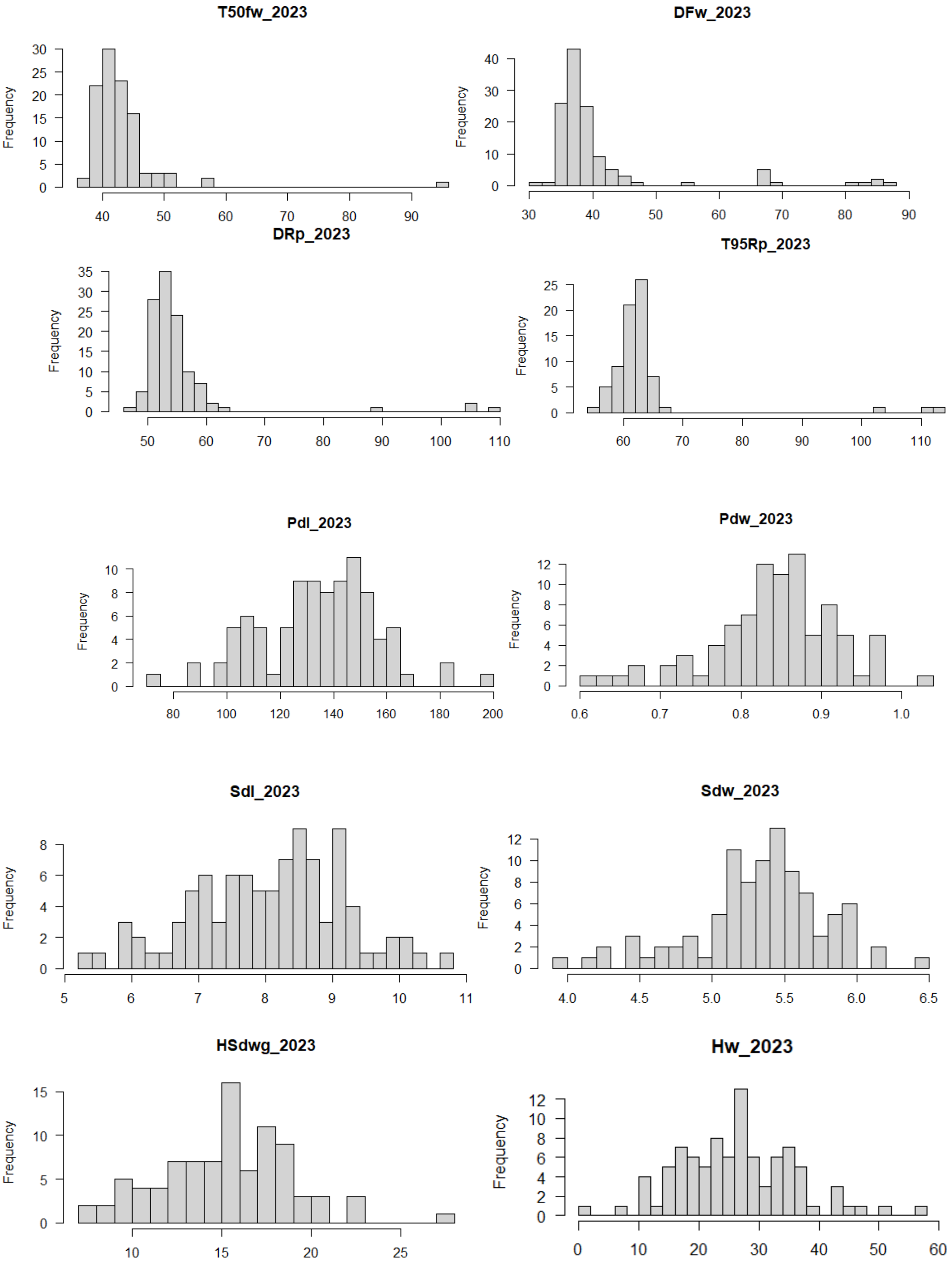

Supplementary Figure 1 : Trait variation in 2023 trial: T50fw:Time to 50% flowering, Dfw: Time to 1st flower, Drp: Time to 1st mature pod, T95rp: Time to 95% pod maturity, Pdl: Pod length, Pdw: Pod width, Sdl: Seed length, Sdw: Seed Width, HSdwg: 100-seed weight, Hw: dry Haulm weight
