# Supplementary material for: Genetic architecture of cowpea domestication: QTL mapping and comparison shed new light on the dual domestication events
Source: G3 (Bethesda). 2025 Oct 17;16(1):jkaf248. doi: 10.1093/g3journal/jkaf248 (PMC12774598; doi:10.1093/g3journal/jkaf248)
Supplement: jkaf248_Supplementary_Data [file jkaf248_supplementary_data.zip › Supplemental_Material_Legends_G3-2025-406133.docx]

**Supplementary Materials**

Supp Figure1: Trait Variation in 2023 trial.

Supp Table 1: ANOVA Tables Trial 2023

Supp Table 2: ANOVA Tables Trial 2024
